# Is there a role for impaired DNA mismatch repair system in the pathogenesis of ameloblastomas? A scoping review

**DOI:** 10.1016/j.jobcr.2026.101477

**Published:** 2026-06-04

**Authors:** Allan Vinícius Martins-de-Barros, Raisa Jordana Geraldine Severino-Lazo, Maria Eduarda de Alencar Barreto, Gilberto Tenório Wanderley Fernandes Lima, Moan Jefter Fernandes Costa, Pedro Henrique Sette-de-Souza, Fábio Andrey da Costa Araújo, Adauto Gomes Barbosa Neto

**Affiliations:** a Universidade de Pernambuco, Campus Arcoverde, Programa de Pós Graduação em Saúde e Desenvolvimeto Socioambiental, Arcoverde, Pernambuco, Brazil; b Universidade de Pernambuco, Campus Santo Amaro (Faculdade de Ciências Médicas), Programa de Pós-Graduação em Ciências da Saúde, Recife, Pernambuco, Brazil; c Universidade de Pernambuco, Campus Santo Amaro (Faculdade de Odontologia de Pernambuco), Programa de Pós-Graduação em Odontologia, Recife, Pernambuco, Brazil; d Universidade de Pernambuco, Campus Santo Amaro (Instituto de Ciências Biológicas), Laboratório Multiusuário em Saúde, Recife, Pernambuco, Brazil

**Keywords:** Odontogenic tumors, Ameloblastoma, DNA repair, DNA mismatch repair, Biomarkers, Tumor

## Abstract

**Background:**

Deficiencies in the DNA mismatch repair (MMR) system are associated with several human neoplasms. However, the role of MMR alterations in ameloblastoma pathogenesis remains poorly understood. This scoping review aimed to synthesize the available evidence on the expression patterns of MMR proteins in ameloblastomas and to discuss their potential biological and clinical implications.

**Methods:**

This scoping review was conducted in accordance with the PRISMA-ScR guidelines and registered in PROSPERO (CRD420251150967). A comprehensive literature search was performed in PubMed/MEDLINE, Web of Science, Embase, and Scopus to address the following question: “What is the expression pattern of DNA mismatch repair system proteins in ameloblastomas?”. Cross-sectional studies, cohort studies, case-control studies, and case series were included, without restrictions on publication date or language. Qualitative and quantitative data were systematically extracted, tabulated, and synthesized.

**Results:**

The search retrieved 97 articles. Five studies met the eligibility criteria, comprising a total of 271 ameloblastoma samples. A relatively consistent pattern of MMR protein expression was observed across studies. Ameloblastomas showed reduced expression of MMR proteins compared with tooth germ controls, with more pronounced alterations in conventional than in unicystic ameloblastomas. Changes in MMR expression were potentially associated with tumor proliferative activity, risk of recurrence, and the presence of BRAF mutations.

**Conclusion:**

Altered expression of MMR proteins appears to be involved in key aspects of ameloblastoma biology, including genomic stability, tumor proliferation, and recurrence risk. Further studies are needed to clarify the potential role of MMR dysregulation as a clinically relevant biomarker in ameloblastomas.

## 1 Introduction

Ameloblastoma is a benign epithelial odontogenic tumor characterized by locally aggressive behavior and a marked tendency for infiltration and destruction of adjacent bone and soft tissues. Owing to its relative frequency among odontogenic tumors and its significant clinical impact, ameloblastoma is widely regarded as the most clinically relevant odontogenic neoplasm.^1^ Despite being histologically benign, the tumor is associated with high recurrence rates, even following radical surgical management, underscoring its complex biological behavior.^2^^,^^3^

Over the past decades, advances in molecular pathology have substantially expanded the understanding of the genetic landscape underlying ameloblastoma tumorigenesis.^4^^,^^5^ Activating mutations involving the mitogen-activated protein kinase (MAPK) pathway, especially the *v-Raf murine sarcoma viral oncogene homolog B1 (BRAF) V600E* mutation, are present in over 65% of all ameloblastomas.^6^^,^^7^ These findings have reinforced the role of oncogenic driver mutations in disease development and have opened new perspectives for targeted therapy. Nevertheless, the contribution of DNA repair mechanisms to tumor initiation, progression, and biological heterogeneity remains poorly understood. Moreover, despite advances in molecular characterization, there are still no robust biomarkers capable of reliably predicting tumor aggressiveness or recurrence risk in patients with ameloblastoma, highlighting the need for further investigation into alternative molecular pathways involved in tumor behavior.^8^^,^^9^

The DNA Mismatch Repair (MMR) System plays a crucial role in correcting base-pairing errors during DNA replication. In human cells, this process is mediated by heterodimeric complexes of the MutS family, including MutS homolog 2 (MSH2), MutS homolog 3 (MSH3), and MutS homolog 6 (MSH6), and the MutL family, including MutL homolog 1 (MLH1), MutL homolog 3 (MLH3), postmeiotic segregation increased 1 (PMS1), and postmeiotic segregation increased 2 (PMS2), which coordinate mismatch recognition, recruitment of endonucleases, and excision of erroneous DNA segments.^10^ Deficiencies in this system, whether due to mutations or epigenetic events, are related to microsatellite instability and increased mutational burden, contributing to the pathogenesis and progression of various neoplasms.^11^^,^^12^

In this context, the evaluation of the expression of MMR proteins in ameloblastomas represents a relevant and largely unexplored avenue for advancing the understanding of their molecular biology. Alterations in DNA repair pathways may influence not only genomic stability but also tumor behavior, recurrence patterns, and response to emerging targeted or immunomodulatory therapies. However, the currently available evidence is limited and heterogeneous, consisting mainly of small observational studies with different immunohistochemical approaches and evaluated markers.

Therefore, a scoping review was considered the most appropriate strategy to map the existing evidence and identify current knowledge gaps in this emerging field. Thus, this paper aimed to systematically synthesize the available evidence regarding the immunohistochemical expression patterns of MMR system proteins in ameloblastomas and discuss their potential role in tumor pathogenesis, biological behavior, recurrence, and possible diagnostic, prognostic, and therapeutic implications.

## 2 Materials and methods

### 2.1 Protocol and registration

This scoping review was structured based on the 5-step method proposed by Arksey and O'Malley^13^: identifying the research question; identifying relevant studies; study selection; charting the data; and collating, summarizing, and reporting results. The review process was conducted in accordance with the recommendations of the Preferred Reporting Items for Systematic Reviews and Meta-Analyses for Scoping Reviews (PRISMA-ScR)^14^ and registered in PROSPERO under registration number CRD420251150967.

### 2.2 Eligibility criteria

Based on the research question: “What is the expression pattern of DNA mismatch repair system proteins in ameloblastomas?”, cross-sectional studies, cohort studies, case-control studies, and case series were included in this review. No restrictions regarding language or publication date were applied. Literature reviews, animal studies, *in vitro* studies, studies presenting duplicate data, as well as studies that did not report data on the expression pattern of MMR proteins in the analyzed samples were excluded.

### 2.3 Search strategy

A systematic search for studies published up to September 14, 2025, was performed in the PubMed/Medline, Web of Science, Embase, and Scopus databases. The search was conducted independently by two authors (AVMB and RJGSV), using the following combination of free terms and MeSH/DeCS descriptors: ("ameloblastoma") AND ("DNA repair" OR "mismatch repair" OR "mismatch" OR "MutS" OR "MutL" OR "mlh1" OR "hmlh1" OR "pms2" OR "hpms2" OR "msh2" OR "hmsh2" OR "msh3" OR "hmsh3" OR "msh6" OR "hmsh6") (Supplementary Table 1). After duplicate removal, the authors screened the publications based on title and abstract, assessing them for eligibility. Disagreements were resolved by a third author (AGBN). Additionally, the reference lists of the selected articles were manually searched by the same authors to identify additional works that might not have been found in the initial database search.

Cohen's kappa coefficient was used to calculate inter-rater agreement during publication inclusion, yielding a level of almost perfect agreement between authors (kappa = 0.90).

### 2.4 Data extraction

Data extraction was performed by two researchers (MJFC and PHSS). The extracted data were classified as quantitative or qualitative, tabulated for comparison, and verified by a third evaluator (FACA), who resolved any disagreements. Throughout this study, data on the following variables were identified and evaluated: authorship; year of publication; study design; sample size; clinicopathological pattern and histological subtype of the tumor; evaluated proteins; primary antibody and immunostaining system used in the immunohistochemical reaction; and immunohistochemical expression profile of MMR proteins, considering positivity/negativity pattern, intensity, and/or quantification.

### 2.5 Data synthesis

The qualitative and quantitative data extracted from the studies were tabulated, analyzed, synthesized, and organized into the following sections for presentation in text and table format: Literature search; Description of the included studies; Expression pattern of MMR proteins in ameloblastomas; Comparison between ameloblastomas and tooth germs; MMR and proliferation index in ameloblastomas; MMR and ameloblastoma prognosis; and MMR and *BRAF* mutations.

## 3 Results

### 3.1 Literature search

**Fig. 1:**
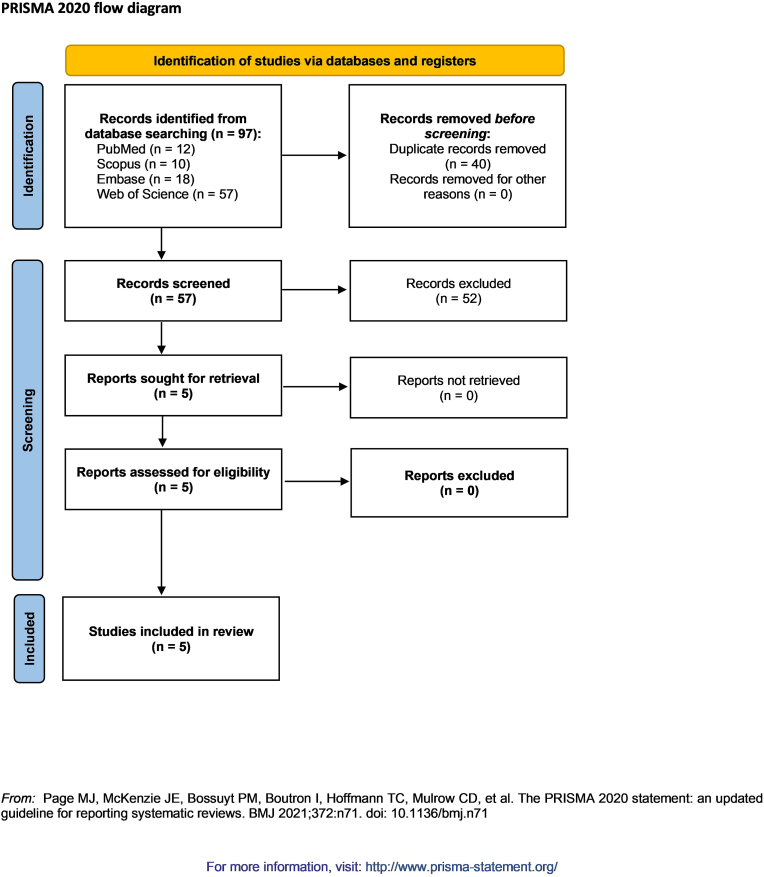
PRISMA-ScR flow diagram of the search and study selection process.

The initial database search retrieved 97 articles. Duplicate references were removed, and subsequently, the remaining titles and abstracts were screened. Five articles were selected for full-text analysis, and all met the eligibility criteria for inclusion in this scoping review. The PRISMA-ScR flow diagram showing the entire article selection process is illustrated in Fig. 1.

### 3.2 Description of the included studies

The detailed description of the studies is available in Table 1, Table 2.

**Table 1:** Detailed description of the studies included in the systematic review.

AUTHOR (YEAR)	STUDY DESIGN	SAMPLE SIZE	SEX	AGE	TUMOR LOCATION	AMELOBLASTOMA TYPE	COMPARISON GROUP
Castrilli et al. (2001)^15^	Cross-sectional	25	NR	Mean: NR Range: NR	NR	Conventional (n = 19) Unicystic (n = 3) Peripheral (n = 3)	None
Amaral-Silva et al. (2017)^16^	Cross-sectional	112^a^	Male (n = 17) Female (n = 17) NR (n = 5)	Mean: 26 Range: 20-30	Mandible (n = 30) Maxilla (n = 9)	Conventional (n = 39)	10 tooth germs
Bologna-Molina et al. (2018)^17^	Cross-sectional	80	NR	Mean: NR Range: NR	NR	Conventional (n = 40) Unicystic (n = 40)	05 tooth germs
Amaral-Silva et al. (2022)^18^	Cross-sectional	10	Male (n = 6) Female (n = 4)	Mean: 34.3 Range: 12 – 79	Mandible (n = 10)	Conventional (n = 10)	None^b^
Pires et al. (2025)^19^	Case-control	44	Male (n = 20) Female (n = 24)	Mean: 39.1 ± 19.8 Range: NR	Mandible (n = 40) Maxilla (n = 4)	Conventional (n = 36) Unicystic (n = 8)	None^c^

NR, not reported.

a The study included 39 cases and a Tissue Microarray (TMA) with 73 viable cases. The TMA cases were not included in the demographic and clinical analysis.

b The tooth germs included as controls in the methylation analysis were not subjected to immunohistochemical expression analysis of MMR proteins.

c The comparison was made between primary and recurrent tumors.

**Table 2:** Summarized data on the expression pattern of DNA mismatch repair system proteins in ameloblastomas.

AUTHOR (YEAR)	MMR PROTEINS ASSESSED	PRIMARY ANTIBODY	METHOD FOR IHC ASSESSMENT	PERCENTILE OF MMR PROTEINS EXPRESSION IN AELOBLASTOMAS	SYNTHESIS OF THE MAIN FINDINGS
Castrilli et al. (2001)^15^	MLH1 MSH2	Anti-MLH1: Clone G168–728 Anti-MSH2: Clone FE11	Qualitative	NR	The immunoexpression pattern of MLH1 and MSH2 proteins in the neoplastic epithelium of ameloblastomas is nuclear, with a predominance of staining in the palisading cells of the basal layer.
Amaral-Silva et al. (2017)^16^	MSH2 MSH3 MSH6	Anti-MSH2: polyclonal Anti-MSH3: polyclonal Anti-MSH6: polyclonal	Manual quantitative, using *ImageJ* software	MSH2: 43,2% MSH3: 59,5% MSH6: 40,9%	MSH2 and MSH6 expression levels were significantly lower in ameloblastomas compared to tooth germs. Increased MMR protein expression levels were associated with the presence of the *BRAF V600E* mutation and tumor recurrence.
Bologna-Molina et al. (2018)^17^	MLH1 MSH2	Anti-MLH1: Clone ES05 Anti-MSH2: Clone FE11	Manual quantitative, using Microsoft Office PowerPoint software	MLH1 Conventional: 59.4 ± 13.5% Unicystic: 62.5 ± 43.4% MSH2 Conventional: 75.8 ± 40.2% Unicystic: 83.3 ± 47.8%	MLH1 and MSH2 expression levels were significantly lower in conventional ameloblastomas compared to unicystic ones and tooth germs. MMR protein expression levels were inversely proportional to Ki67 expression levels.
Amaral-Silva et al. (2022)^18^	MSH2 MSH3 MSH6	Anti-MSH2: polyclonal Anti-MSH3: polyclonal Anti-MSH6: polyclonal	Automated quantitative, using *Nuclear V9 Algorithm* software	MSH2: 47,68 ± 26,69, 7,20 – 83,17 MSH3: 25,70 ± 12,82, 2,25 – 44,25 MSH6: 12,69 ± 5,44, 4,76 – 22,75	A strong negative correlation was observed between MSH2 and MSH6 protein expression levels and methylation in their respective genes.
Pires et al. (2025)^19^	MLH1 MSH2	Anti-MLH1: Clone G168-15 Anti-MSH2: Clone G219-1129	Manual quantitative, using *ImageJ* software	NR	MLH1 and MSH2 expression levels were categorized as high (>50%) or low (≤50%). Low MMR protein expression levels were associated with tumor recurrence.

IHC, immunohistochemistry. MMR, Mismatch Repair. NR, not reported.

Five studies published between 2001 and 2025 were included.^15^, ^16^, ^17^, ^18^, ^19^ All studies presented an observational study design, with four being cross-sectional and one case-control. In total, the studies analyzed 271 ameloblastoma cases, including the clinicopathological variants: conventional, unicystic, and peripheral.

### 3.3 Expression pattern of MMR proteins in ameloblastomas

All the included studies used immunohistochemistry to evaluate the expression pattern of MMR proteins in the samples. Two studies evaluated both MutSα and MutSβ complexes (proteins MSH2, MSH3, and MSH6),^16^^,^^18^ while the other three studies evaluated only one protein of the MutL (MLH1) and one protein of the MutS (MSH2) complexes.^15^^,^^17^^,^^19^ The primary antibodies used in the immunohistochemical reaction varied considerably among studies.

For the evaluation of MMR protein expression, a quantitative method was used in most of the analyzed studies. In four studies^16^, ^17^, ^18^, ^19^, the percentage of neoplastic cells with positive immunostaining was quantified manually or automatically. However, Castrilli et al. (2001)^15^ performed only a descriptive qualitative analysis of the observed immunostaining pattern.

The expression pattern of MMR proteins in ameloblastomas was similar in all included studies. The MLH1, MSH2, MSH3, and MSH6 proteins presented exclusively nuclear expression, with variable intensity. There was a predominance of positive staining in the palisading columnar cells, resembling ameloblasts, located in the outermost layers of the neoplastic nests, while the more central areas, which present cells resembling the stellate reticulum of the enamel organ, were generally negative. Similarly, central areas with squamous differentiation or granular cells were also negative. The stroma, composed of connective tissue of variable density, occasionally exhibited focal immunostaining.

There was great variability in the quantification of MMR proteins among studies and among different samples within the same study. Conflicting findings were particularly observed for MSH2 expression, with Bologna-Molina et al. (2018)^17^ reporting relatively high expression levels (75.8 ± 40.2% to 83.3 ± 47.8%), whereas Amaral-Silva et al. (2017)^16^ and Amaral-Silva et al. (2022)^18^ demonstrated lower expression values (43,2% and 47,68 ± 26,69%, respectively), as detailed in Table 2. These discrepancies may be related to differences in sample composition, primary antibodies, and immunohistochemical quantification methods, reinforcing the heterogeneity of the currently available evidence.

No differences were observed in the protein expression pattern among the different histological subtypes of ameloblastomas. Also, no associations were described between the expression levels of MMR proteins and other clinical characteristics, such as sex, age, anatomical location of the tumor, tumor size, and imaging patterns.

### 3.4 Comparison between ameloblastomas and tooth germs

In only two studies,^16^^,^^17^ the expression of MMR proteins observed in ameloblastomas was compared with that observed in normal odontogenic epithelium originating from human tooth germs. The expression levels of MLH1, MSH2, and MSH6 proteins were significantly lower in ameloblastomas compared to tooth germs, suggesting a possible deficiency of the MMR system in these tumors. Furthermore, Bologna-Molina et al. (2018)^17^ observed that this difference was more marked for solid/conventional than for unicystic ameloblastomas.

### 3.5 MMR and proliferation index in ameloblastomas

Two studies evaluated the correlation between the expression levels of MMR system proteins and cell proliferation, measured by the nuclear marker Ki67. While Amaral-Silva et al. (2017)^16^ did not observe a correlation between the expression of the analyzed proteins and cell proliferation indices. Bologna-Molina et al. (2018)^17^ observed that the expression levels of MLH1 and MSH2 proteins were inversely proportional to Ki67 expression levels, indicating higher proliferation in tumors with lower MMR system activity.

### 3.6 MMR and ameloblastoma prognosis

Amaral-Silva et al. (2017)^16^ and Pires et al. (2025)^19^ also investigated possible associations between MMR protein expression and the prognosis of ameloblastomas, with divergent results. Amaral-Silva et al. (2017)^16^ reported an association between the simultaneous overexpression of MutS complex proteins (MSH2, MSH3, and MSH6) and the occurrence of tumor recurrence, without, however, a statistically significant difference in disease-free survival. On the other hand, Pires et al. (2025) identified that low expression levels (≤50%) of MLH1 and MSH2 in ameloblastomas may increase the risk of tumor recurrence, with a direct impact on disease-free survival curves.

### 3.7 MMR and BRAF mutations

Furthermore, only Amaral-Silva et al. (2017)^16^ evaluated the relationship of MSH2, MSH3, and MSH6 expression levels with the *BRAF V600E* mutation. In this study, the authors observed that ameloblastomas presenting the mutation exhibited a statistically significant increase in the expression of MutSβ heterodimer (MSH2 and MSH3), compared to wild-type *BRAF* ameloblastomas.

## 4 Discussion

Despite its fundamental role in maintaining genomic stability,^11^ the investigation of the DNA MMR system in odontogenic lesions remains a poorly explored field. The vast majority of studies addressing MMR protein expression in human neoplasms focus on gastrointestinal, gynecological, and central nervous system tumors, where MMR deficiency is well established as a driver of genomic instability and clinical behavior.^20^, ^21^, ^22^, ^23^ To the best of our knowledge, this is the first review specifically dedicated to synthesizing and critically appraising the available evidence on MMR protein expression in ameloblastomas.

MMR system deficiency represents a canonical mechanism of genomic instability, classically described in hereditary cancer predisposition syndromes, such as Lynch syndrome, but currently also established as an important biomarker in several sporadic malignancies.^24^^,^^25^ In these scenarios, loss of MMR function is associated with microsatellite instability and increased mutational burden, and presents clinically relevant prognostic and therapeutic implications.^26^^,^^27^

In ameloblastomas, although the complete loss of MMR protein immunoexpression has not been reported, the reviewed studies consistently demonstrated reduced expression of key MMR proteins, particularly MLH1, MSH2, and MSH6, when compared with normal odontogenic tissues. These findings suggest that at least a partial impairment of the MMR system may contribute to ameloblastoma pathogenesis.

Notably, mutations in genes encoding core MMR proteins have not been reported in ameloblastomas,^17^ indicating that reduced protein expression may result from alternative regulatory mechanisms. Epigenetic modulation, especially promoter hypermethylation, emerges as a plausible explanation. Amaral-Silva et al. (2022)^18^ observed a negative correlation between gene methylation and MSH2 and MSH6 expression in ameloblastomas, reinforcing the hypothesis that epigenetic silencing may contribute to functional attenuation of the MMR system. This finding reinforces the hypothesis that epigenetic alterations may act in the inactivation of the MMR system in ameloblastomas, contributing to the occurrence of replication errors. Similar mechanisms have been extensively documented in esophageal, colorectal, endometrial, and sebaceous neoplasms, in which MLH1 promoter methylation is a frequent cause of MMR deficiency.^28^, ^29^, ^30^ However, epigenetic studies involving ameloblastomas and other odontogenic tumors remain scarce, limiting a broader understanding of the molecular mechanisms underlying these lesions.

An additional layer of complexity arises from the interaction between oncogenic signaling pathways and DNA repair mechanisms. Activating mutations in the *BRAF* gene have been shown to induce epigenetic alterations affecting DNA repair genes. Oliveira da Silva et al. (2023)^31^ identified that the methylation status of the MLH1 gene was concordant with the mutational status of the *BRAF* gene in 90% of analyzed colorectal cancer cases with MLH1/PMS2 deficiency. Interestingly, Amaral-Silva et al. (2017)^16^ also identified alterations in the expression pattern of MSH2 and MSH3 in ameloblastomas with the *BRAF V600E* mutation. Collectively, these findings suggest that MAPK pathway activation may influence DNA repair capacity through epigenetic modulation, facilitating the accumulation of molecular alterations necessary for tumor progression and maintenance.

Regarding biological behavior, the studies included in this review yielded heterogeneous and, at times, conflicting results concerning the association between MMR protein expression, cell proliferation, tumor recurrence, and disease-free survival. In colorectal carcinoma, MMR deficiency is typically associated with high proliferative activity and mutational load, yet paradoxically confers a more favorable prognosis, largely attributed to enhanced tumor immunogenicity.^27^^,^^32^^,^^33^ This contrast highlights that the biological consequences of impaired DNA repair are highly context-dependent. In ameloblastomas, the clinical implications of altered MMR expression remain unclear, and the discrepancies reported by Amaral-Silva et al. (2017)^16^ and Pires et al. (2025)^19^ may reflect true biological heterogeneity, methodological differences, or both.

Although immunohistochemistry remains the standard method for assessing MMR protein expression,^10^, ^11^, ^12^ most included studies relied exclusively on this approach, without integration with contemporary molecular techniques such as methylation profiling, next-generation sequencing, or other functional molecular assays. In addition, substantial heterogeneity was observed among studies regarding antibody selection, staining protocols, scoring systems, cut-off definitions, and quantification methods. The evolution of immunohistochemical analysis over time, from predominantly qualitative assessment to digital and automated quantitative approaches, may also partially explain discrepancies among findings. Although international criteria for MMR evaluation by immunohistochemistry are currently available for several tumors, no standardized parameters have been established for odontogenic tumors, particularly due to the absence of studies investigating MMR proteins in lesions other than ameloblastoma.^34^

Additionally, the limited number of available studies, small sample sizes, retrospective observational designs, and moderate methodological bias restrict the robustness of the current evidence. The heterogeneity of clinicopathological data and the lack of standardized outcome measures also preclude definitive correlations between MMR protein expression and clinical behavior, recurrence risk, or prognostic outcomes in ameloblastomas.

These gaps reinforce the need for additional research evaluating, in an integrated manner, the different molecular mechanisms that may alter the expression pattern of MMR proteins in ameloblastomas and other odontogenic tumors. A more comprehensive understanding of the mechanisms underlying MMR dysregulation in these lesions may clarify its role in tumor pathogenesis and progression, as well as support the identification and validation of biomarkers with diagnostic, prognostic, and therapeutic relevance.

## 5 Conclusions

In conclusion, this review synthesized the available evidence on the expression of DNA MMR proteins in ameloblastomas, revealing that reduced or altered levels of MLH1, MSH2, and MSH6 may be implicated in tumor biology, particularly regarding cell proliferation, risk of recurrence, and association with activating mutations in the *BRAF* gene. Further studies are needed to understand how these molecular alterations impact the pathogenesis and prognosis of ameloblastomas, as well as to evaluate their potential as clinical biomarkers.

## Informed consent statement

Not applicable for review studies.

## Institutional review board statement

Not applicable for review studies.

## Declaration of competing interest

The authors declare that they have no known competing financial interests or personal relationships that could have appeared to influence the work reported in this paper.
