# Supplementary material for: Is there a role for impaired DNA mismatch repair system in the pathogenesis of ameloblastomas? A scoping review
Source: J Oral Biol Craniofac Res. 2026 Jun 4;16(4):101477. doi: 10.1016/j.jobcr.2026.101477 (PMC13266097; doi:10.1016/j.jobcr.2026.101477)
Supplement: Multimedia component 1 [file mmc1.docx]

**Supplementary Table 1.** Full search strategies according to each database.

| **DATABASE** | **SEARCH SYNTAX** |
| --- | --- |
| PubMed/MEDLINE | ("ameloblastoma"[All Fields]) AND ("DNA repair"[All Fields] OR "mismatch repair"[All Fields] OR "mismatch"[All Fields] OR "MutS"[All Fields] OR "MutL"[All Fields] OR "mlh1"[All Fields] OR "hmlh1"[All Fields] OR "pms2"[All Fields] OR "hpms2"[All Fields] OR "msh2"[All Fields] OR "hmsh2"[All Fields] OR "msh3"[All Fields] OR "hmsh3"[All Fields] OR "msh6"[All Fields] OR "hmsh6"[All Fields]) |
| Web of Science | ALL=(ameloblastoma) AND ALL=("DNA repair" OR "mismatch repair" OR mismatch OR MutS OR MutL OR mlh1 OR hmlh1 OR pms2 OR hpms2 OR msh2 OR hmsh2 OR msh3 OR hmsh3 OR msh6 OR hmsh6) |
| Embase | (ameloblastoma:all) AND ('DNA repair':all OR 'mismatch repair':all OR mismatch:all OR MutS:all OR MutL:all OR mlh1:all OR hmlh1:all OR pms2:all OR hpms2:all OR msh2:all OR hmsh2:all OR msh3:all OR hmsh3:all OR msh6:all OR hmsh6:all) |
| Scopus | ALL ( ameloblastoma ) AND ALL ( "DNA repair" OR "mismatch repair" OR mismatch OR MutS OR MutL OR mlh1 OR hmlh1 OR pms2 OR hpms2 OR msh2 OR hmsh2 OR msh3 OR hmsh3 OR msh6 OR hmsh6 ) |
